# The Intention and Uptake of Colorectal Cancer Screening after a Brief Health Education Program in a Malaysian Primary Care Setting: A Population-Based Study

**DOI:** 10.31557/APJCP.2021.22.11.3475

**Published:** 2021-11

**Authors:** Mei Wai Chan, Kooi Yau Chean, Siti Fatimah Kader Maideen, Fei Ping Kow

**Affiliations:** 1 *Department of Family Medicine, RCSI&UCD Malaysia Campus, Georgetown, Penang, Malaysia. *; 2 *Department of Public Health, RCSI&UCD Malaysia Campus, Georgetown, Penang, Malaysia. *; 3 *Department of Outpatient, Jalan Angsana Health Clinic, Ayer Itam, Penang, Malaysia. *

**Keywords:** Colorectal cancer, screening, health education, primary health care, Malaysia

## Abstract

Colorectal cancer (CRC) is the most common cancer in men and the second most common cancer in women in Malaysia. A major challenge for CRC screening programs is to improve the screening participation rates. In Malaysia, the most critical barrier to the uptake of CRC screening is the lack of patient awareness. This study aimed to determine the intention and the uptake of CRC screening, and to explore the related motivators and barriers after raising awareness with a brief health education.

**Methods:**

An analytical cross-sectional study was conducted in a government health clinic of Penang from March to August 2019. Asymptomatic clinic attendees aged 50-75 years who had no prior awareness of CRC screening were recruited by systematic random sampling technique. Participants first received a standardised one to one health education, followed by an interview using a standardised questionnaire to assess their CRC screening intention and the relevant motivators and barriers. A submission of a sample for immunochemical faecal occult blood test (iFOBT) was considered as an uptake of the CRC screening.

**Results:**

A total of 546 participants participated in this study. The mean age of the participants was 62.8 (SD=6.36). Majority of them were females (57.3%), Chinese (78.6%), who had attained primary or higher education (92.0%) and had comorbidities (87.0%). After a brief health education, 231 participants (42.3%) agreed to undergo iFOBT. The actual screening uptake rate in this study was 28%. Perceived benefit of the test (84.4%) was the most common motivators, while self-perceived non-vulnerability was the biggest impediment to CRC screening intention. Physicians’ recommendation was the perceived most effective way in raising CRC awareness.

**Conclusion:**

Participants prefer physicians to provide health education. Standardised brief health education is inadequate to stimulate CRC screening adherence. Future interventions will require in-depth understanding of patients’ beliefs, risk perception, and affective responses.

## Introduction

Colorectal cancer (CRC) is the third most prevalent cancer and the second highest in cancer mortality globally (Bray et al., 2018). Developed countries have about 3-4 folds higher CRC incidence than developing countries (Bray et al., 2018; Rawla et al., 2019). The rates remain the highest in Australia, Japan and the USA but in a stabilizing or decreasing trend (Arnold et al., 2017). On the contrary, the incidence and the mortality of CRC are rising rapidly in developing countries that are adopting a more “western” lifestyle (Arnold et al., 2017; Rawla et al., 2019). In Malaysia, CRC is the most common cancer in men(14.8%) and second most common cancer in women(11.1%) (Ministry of Health Malaysia, 2019) and the incidence is rising.

The long preclinical phase of CRC allows an opportunity for early detection. CRC screening is recommended for population aged 50-75 years using immunochemical feacal occult blood test (iFOBT)(Sung et al., 2015). Widespread CRC screening has been proven to be cost effective (Lansdorp-Vogelaar et al., 2011; Ran et al., 2019) in reducing its incidence and mortality. The implementation of CRC screening program varies among countries across the world due to variation of the incidence of CRC, economy status of the country, health care structure and infrastructure to support the program.(Schreuders et al., 2015). In Malaysia, there is currently no population-based screening for CRC. A national pilot program on CRC screening has been implemented since 2012 at selected government clinics. In this program, the public are screened using iFOBT in the clinics. Those tested positive will then be referred to a hospital for verification colonoscopy. In the current practice, the screening is done opportunistically either based on the doctor’s recommendation or patient’s request. Primary care physicians face challenges to provide patient education within the constraint of busy clinical settings. 

In a multicenter study involving 14 Asia-Pacific countries (Koo et al., 2012), Malaysia has the second lowest rate of previous CRC testing (3%) after India. Eighty percent of Malaysian did not know about CRC screening test and more than half the population did not know about the symptoms and risk factors of CRC. Additionally, 65%of CRC in Malaysia was detected at stage III and IV(Arunah et al., 2020). Hence, the overall survival of Malaysian CRC patients for comparable stage of CRC is lower than achieved in developed countries (Veettil et al., 2017).

In view of the poor knowledge of CRC and low CRC screening rate in the population, this study aimed to determine the rate of intention and the uptake of CRC screening after a brief one-to-one health education in a primary care setting. In addition, we explored the motivators and barriers to undergo CRC testing and their preferred methods in promoting CRC screening. Understanding these factors is important to modify specific factors to increase the uptake of CRC screening.

## Materials and Methods

*Study design and participants*

This is an analytical cross-sectional study. The study was conducted at the Jalan Angsana Health Clinic (formerly known as Bandar Baru Air Itam Health Clinic) from March 2019 to August 2019. Jalan Angsana Health Clinic is a government primary care clinic situated in a residential neighbourhood, lies 6 kilometres from the city centre (Georgetown) in the state of Penang, Malaysia. In 2019, the estimated population served by this clinic was 86685 individuals with 22140 (25.5%) aged between 50 to 75 years of age.

The inclusion criteria were Malaysian nationality, aged between 50 to 75 years and who were unaware of CRC screening during initial assessment. The exclusion criteria were those who had history of colon cancer, previous CRC screening, or feeling too ill on the recruitment day. A written consent was obtained from the eligible participants after explaining the purpose and the procedure of the study. Additionally, participants were given assurance of data confidentiality and informed of their right to withdraw from the study at any time. 

The sample size was calculated using Openepi Version 3.0 sample size calculator (Dean et al., 2013). By taking 32.3% proportion of intention for colorectal screening among those who perceived having risk of CRC (Naing et al., 2014) and an estimated difference of 15% in the current setting, 90% power and a significance level set at 5%, the sample size required for this study was 472 patients. Taking into consideration of 50% non-response rate, the final sample size for this study was 708 patients.

Participants were recruited using a systematic random sampling method. The estimated total number of eligible participants was 3000 (estimated 50 participants over a period of 60 days). A sampling interval of five was obtained by dividing the total number of eligible participants with the number of required sample size. For the selection of first participant for the day, a simple random sampling using the lottery method was used. Number 1 to 10 was included in the ballots and one number was randomly picked from the ballots. Then at every 5th interval, one participant was selected until the required sample size was met. 

*Data collection*

Two recent medical graduates were trained to provide brief patient education and to interview participants for data collection. Participants were first given a 5-10- minutes standardized brief health education which comprised of the epidemiology, risk factors, signs and symptoms of CRC, the importance of the CRC screening and explanation iFOBT screening test. This was followed by an interview using a standardized structured questionnaire, which was developed based on the information from the local literatures (Hilmi et al., 2010; Koo et al., 2012; Wong et al., 2013). Each participant was asked if they would be willing to be screened for CRC. Motivating factors behind the willingness and barriers behind the refusal were asked accordingly. Participants who intended to undergo iFOBT were then given a container for stool sample and they were asked to submit the stool sample within 2 weeks. For those who did not send their samples within the two weeks were considered as non-response for the screening test. The stool sample was tested using iFOBT kit by a laboratory assistant in the clinic. A follow-up appointment within a month was given to the participants to review the test result. A reminder call was also given within a week to the defaulters. For those who have positive iFOBT result, they were given referral letter for a colonoscopy. 

*Study measures*

The intention to screen and the uptake of iFOBT screening test were assessed. The intention to screen was assessed immediately after the brief health education. Additionally, barriers and motivators for the intention to undergo iFOBT were also assessed. The uptake of iFOBT screening test was measured 2 weeks later. The dependent variables in this study were intention to screen for CRC and actual uptake of the screening. While the independent variables were socio-demographics, the motivators, and barriers for CRC screening. 

*Data analysis*

Data analysis was performed with IBM SPSS Statistical Software, version 25.0. Out of the 546 participants, there were missing data from two participants on perceived effective medium in promoting CRC screening. The incomplete data was excluded from the individual variable analysis.

We first examined descriptive statistics of participants using frequencies and proportions. Numerical variables were displayed as mean and standard deviation, whereas categorical variables were displayed as frequency and percentage. Data was analyzed using complete cases (pair-wise deletion). For each pair of variables, data was analysed based on analysis by analysis. Statistical tests were two-tailed with a significant level set at 0.05. Independent t-test was used to determine the mean difference in age and the intention for CRC screening while Chi-square test was used to determine the association between the categorical variables.

## Results

**Figure 1:**
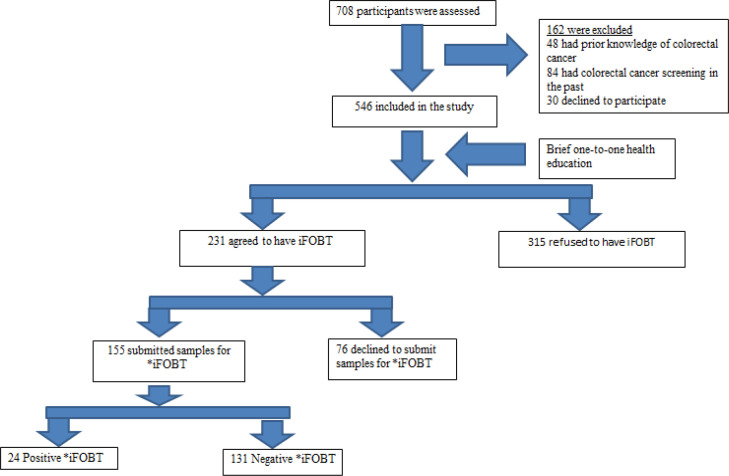
Study flow. *iFOBT, immunochemical feacal occult blood test

Figure 1 shows the flow chart of the study. We approached 708 clinic attendees according to the sampling strategies. A total of 162 attendees were excluded during screening. Of these, 132 (18.6 %) attendees were not eligible because of prior knowledge of CRC screening (n=48) or had been screened for CRC in the past (n=84). The remaining 30 attendees declined to participate in the study. 546 (77.1%) eligible participants were recruited into the study. After a brief health education, 42.3% of the participants (n=231) agreed to undergo iFOBT and subsequently, 67.1% of them (n=155) submitted a specimen for testing.

*Sample characteristics*

The socio-demographic characteristics of the study participants are provided in Table 1. The mean age of the participants was 62.8 (SD=6.36). Majority of the participants were women (57.3%), of Chinese ethnic group (78.6%), married (88.6%) and those who had attained higher than primary education (55%). Most participants (93.2%) live within 5km from the clinic. 

*Factors influencing the intention of CRC screening*

Slightly less than half of the participants expressed willingness to undergo the CRC screening test after a brief health education. Table 2 shows the descriptive analysis of factors influencing the intention to participate in CRC screening. The biggest motivating factor was self-perceived benefit of the test (80.1%). However, among the refusals (57.7%), the most common reason to refuse the test was self-perceived “good health” (48.9%), followed by “lack of time” (22.2%) and “anxious about the outcome of the test” (12.7%). Physicians (34.7%) and health campaign (18.9%) were perceived to be the most effective medium in promoting CRC screening.

*The association between socio-demographic characteristics and screening intention for CRC*

Table 3 shows the association between socio-demographic characteristics and the intention to screen among the participants Being younger, unemployed, having lower education level, or without comorbidity were more likely to agree to CRC screening (p≤0.05). There were no significant association between socio-demographic characteristics and the actual uptake of iFOBT, except for Chinese ethnicity (Table 4).

**Table 1:** Socio-Demographic Characteristics of Study Participants (N=546)

Socio-demographic characteristics	Frequency (Percentage)
Gender	
Male	233 (42.7)
Female	313 (57.3)
Ethnicity	
Malay	34 (6.2)
Chinese	429 (78.6)
Indian	81 (14.8)
Others	2 (0.4)
Marital status	
Single	38 (7.0)
Married	484 (88.6)
Divorced	13 (2.4)
Widowed	11 (2.0)
Highest education level	
No formal education	44 (8.0)
Primary	202 (37.0)
Lower secondary	154 (28.2)
Upper secondary	109 (20.0)
Tertiary	37 (6.8)
Employment status	
Employed	205 (37.5)
Unemployed	341 (62.5)
Smoking status	
Smoker	62 (11.4)
Non-smoker	406 (74.4)
Ex-smoker	78 (14.2)
With comorbidity	
Yes	475 (87.0)
No	71 (13.0)
Distance to nearest health care centre	
Less than 5km	509 (93.2)
5km to 10km	31 (5.7)
10 km to 20 KM	2 (0.4)
More than 20km	4 (0.7)

**Table 2:** Factors Influencing the Intention to Participate in Colorectal Cancer Screening

Reasons that motivate intention to undergo	Frequency (percentage)
screening (n=231)	
Families/relatives/friends with colon cancer	6 (2.6)
Physician’s recommendation	24 (10.4)
Presence of bowel symptoms	6(2.6)
The test is beneficial to me	185 (80.1)
The test is free	9 (3.9)
Others	1 (0.4)
Reasons for refusal of screening (n=315)	
I am healthy	154 (48.9)
I feel anxious or embarrassed to do the test	18 (5.7)
I feel anxious to know the outcome of the test	40 (12.7)
The test is not important to me	14 (4.5)
I have no time	70 (22.2)
Others	19 (6.0)
Perceived effective medium in promoting colorectal cancer screening (n=544)	
Physicians	189 (34.7)
Nurses/Medical assistants	12 (2.2)
friends	44 (8.1)
Newspapers/magazines	57 (10.5)
Television	64 (11.8)
Social media	59 (10.9)
Health campaign	103 (18.9)
Educational brochure	16 (2.9)

**Table 3:** The Association between Socio-Demographic Characteristics with Intention to Undergo for Colorectal Cancer Screening (N=546)

Socio-demographic characteristics	Intention to undergo screening		OR (95% Confidence Interval)
	Yes (n=231) n (%)	No (n=315) n (%)	
Age (Mean ± SD)	61.92 ± 6.42	63.44 ± 6.25	Mean difference -1.52 ((- 2.59) - (-0.439))
Gender			
Male	105 (45.5)	128 (44.6)	0.82 (0.58-1.16)
Female	126 (54.5)	187 (59.4)	
Ethnicity			
Chinese	179 (77.7)	250 (79.2)	1.117 (0.740-1.687)
Non-Chinese	52 (22.3)	65 (20.8)	
Marital status			
Single/widow	24 (10.4)	38 (12.1)	1.183 (0.688-2.034)
Married	207 (89.6)	277 (87.9)	
Highest education level			
Primary and below	86 (37.2)	160 (50.8)	1.740 (1.231-2.461)
Secondary and above	145 (62.8)	155 (49.2)	
Employment status			
Unemployed	132 (57.1)	209 (66.3)	1.479 (1.042-2.099)
Employed	99 (42.9)	106 (33.7)	
Smoking status			
Smoker/ex-smoker	60 (26.0)	80 (25.4)	1.03 (0.70-1.52)
Non-smoker	171 (74.0)	235 (74.6)	
With comorbidity			
Yes	193 (83.5)	282 (89.5)	0.59 (0.36-0.98)
No	38 (16.5)	33 (10.5)	

**Table 4:** The Association between Socio-Demographic Characteristics with Actual Uptake of the Screening Test (N=231)

Socio-demographic characteristics	Actual uptake of the test		OR (95% Confidence Interval)
	Yes (n=155) n (%)	No (n=76) n (%)	
Age (Mean ± SD)	62.17 ± 6.76	61.42 ± 5.66	Mean difference 0.75 (- 1.02 – 2.56)
Gender			
Male	76 (49.0)	29 (38.2)	1.56 (0.89-2.73)
Female	79 (51.0)	47 (61.8)	
Ethnicity			
Chinese	131 (84.5)	48 (63.2)	3.184 (1.683-6.024)
Non-Chinese	24 (15.5)	28 (36.8)	
Marital status			
Single/widow	15 (9.7)	9 (11.8)	0.80 (0.33-1.92)
Married	140 (90.3)	67 (88.2)	
Highest education level			
Primary and below	59 (38.1)	27 (23.5)	01.16 (0.63-1.97)
Secondary and above	96 (61.9)	49 (64.5)	
Employment status			
Unemployed	89 (57.4)	43 (56.6)	1.04 (0.60-1.80)
Employed	66 (42.6)	33 (43.4)	
Smoking status			
Smoker/ex-smoker	42 (27.1)	18 (23.7)	1.20 (0.63-2.26)
Non-smoker	113 (72.9)	171 (76.3)	
With comorbidity			
Yes	130 (83.9)	63 (82.9)	1.07 ( 0.52-2.24)
No	25 (16.1)	13 (17.1)	

## Discussion

This study presented the intention and actual uptake rate of CRC screening. The intention to screen for CRC was 42.3% which is comparable to two other similar Malaysian studies, which are at 38% (Koo et al., 2012) and 39% (Hilmi et al., 2010). Nevertheless, these figures are lower than the neighbouring countries, such as Singapore (62%) (Yong et al., 2016) and Thailand (74.1%) (Koo et al., 2012; Saengow et al., 2015). The intention rate in this study was much lower when compared to the study done in the urban cities of Malaysia, which reported about 80% of participants willing to screen for CRC (Mohd Suan et al., 2015). Such difference in the intention rate could be due to the latter study was done in eight urban cities, which was most probably represented by the more educated participants who might have better understanding of CRC. This study found that despite the provision of one-to-one brief health education, the actual uptake of CRC screening was only 28%. Literature has reported that the most critical barrier to the uptake of CRC screening is the lack of patient knowledge or education (Koo et al., 2012; Azeem E, 2016). For CRC screening with faecal occult blood test (FOBT), one-to-one education has been a recommended community-based intervention for improving screening rates. (Community Preventive Services Task Force, 2012; Sabatino et al., 2012). In addition, physician is the most preferred medium to promote CRC screening (Sung et al., 2008; Hilmi et al., 2010). Therefore, by providing a brief health education to patients in primary care setting would ideally convince patients to undergo FOBT, but the results from this study indicate that more than half of the participants refused FOBT despite one-to-one health education. In addition, one third of those agreed to undergo the screening test failed to submit a stool sample. This is far below from the target recommended by WHO, that is to screen over 70% of population at risk.(World Health Organisation, 2007) 

The main motivator to agree to the CRC screening was self-perceived benefit. On the other hand, the main reason of refusal for CRC screening was self-perceived non-vulnerability, that is “I am healthy”. Similar reason of refusal was also found in Singapore and Thailand (Saengow et al., 2015; Sung et al., 2015; Yong et al., 2016) where participants have misunderstandings or misconceptions of the screening purposes. A study by Tu (Tu et al., 2006) found out that using a clinic-based, culturally and linguistically appropriate intervention was able to improve FOBT screening among the Chinese in the USA because some participants may be resistant to the standardized brief education. We therefore need to understand and address the misconception and risk perception of our patients in CRC screening. This can be challenging especially in Malaysia due to its multiracial and multicultural society. Hence, a qualitative study to look deeper into reasons of refusal locally would be useful. Besides, National Health Morbidity Survey 2015 reported that the adult health literacy level among the Malaysian population was worryingly low with 93.4% of its population has limited health literacy (Abdullah et al., 2019). There is a correlation for the association of inadequate health literacy and lower cancer screening rates. (Oldach and Katz, 2014). 

We found those who are younger or unemployed, with no comorbidity or have lower educational level are more receptive to CRC screening. Studies (Gimeno García, 2012; Su et al., 2013) have reported that those who are unemployed and with lower educational level are associated with poor participation in the CRC screening. So, this subgroup potentially is more receptive to intervention that promote CRC screening. The low awareness or difference in personal health seeking behaviour among the elderly might have further contributed to the lower intention for CRC screening among the older participants. Furthermore, having a disease is a motivator in engaging patients in a screening program (Yusoff et al., 2012) , although this is inconsistent to the study by Gimeno (Gimeno García, 2012). Previous studies from Malaysia reported that Chinese has lower awareness(Su et al., 2013) or negative perception on CRC screening (Hilmi et al., 2010) when compared to non-Chinese. This association however is not seen in the current study. CRC screening is not a population- based program in Malaysia. Hence, more intervention needs to be put in for the older, employed, higher educated population and with comorbidities in CRC screening.

To date, there has been no local study that assess the actual uptake on CRC screening following patient health education. Most studies assume willingness to screen implied uptake of screening. (Hilmi et al., 2010; Koo et al., 2012). This study found one third of participants failed to submit a sample of the test despite verbally agreed to participate in the screening program. Similar findings in a study from Singapore Foo et al., (2012) reported that more than half of their participants did not submit a FOBT sample despite agreeing to the test. Participants who “fear of diagnosis” were less likely to submit a sample (Tu et al., 2006). A study conducted in Hong Kong showed that high perceived behavioural control, high behavioural intention for screening and positive attitudes towards CRC screening, could enhance CRC screening uptake (Huang et al., 2020). Therefore, better understanding of patients’ affective response towards CRC screening is an important strategy to focus on when promoting CRC screening program. A review among the Chinese showed that having health insurance coverage, higher education level, better knowledge in the disease, and physician’s recommendation positively influence one in CRC screening program (Leung et al., 2016). A mixed method study examining the cancer screening uptake in Hong Kong revealed that low knowledge on disease, misconception, lack of access to health information, poor access to screening services and cultural barriers were impediment to screening uptake (So et al., 2020). Therefore, addressing those factors are essential to reduce the risk, morbidity and mortality of CRC.

In this study, physicians’ recommendation was perceived to be the most effective way in delivering the CRC awareness. Physician’s recommendation is a uniform predictor of screening behaviour in all countries of Asia Pacific region (Koo et al., 2012). A study from Hong Kong found family physician ‘s recommendation increases the likelihood of complying with CRC screening by 21- fold (Sung et al., 2008). A study from Malaysia (Hilmi et al., 2010) found that recommendation from physicians was the strongest predictive factor for the willingness to participate in a screening program. Similarly, higher participation rate in Japan and Australia was associated with higher physician recommendation (Wong et al., 2013). Hence, one of the first important step in making the CRC screening a success is to encourage our primary care physicians to promote CRC screening to the patients. Besides, the community should also be well informed about the existence of CRC screening program in the primary health care facilities (Arunah et al., 2020). 

A randomized controlled trial from Australia demonstrated that primary care physician intervention consisting of a combination of printed information on CRC screening advice and face-to-face primary care physician endorsement significantly increased the FOBT screening uptake among those with average risk of CRC (Dodd et al., 2019). Hence, this shows that physicians’ recommendations are very crucial to increase the awareness on CRC and its screening.

There are a few strengths worth mentioning in this study. First, the use of probabilistic sampling increases the generalization of the study findings to the general population in Air Itam, Penang. Second, the use of accurate and reliable screening tool, the iFOBT, increases the validity of the study. Lastly, the large sample size in this study increases the power of the study. However, this is a single-centre study and therefore the external validity of the findings may be limited. 

Although a brief standardized education session can increase patients’ knowledge in CRC, it is however inadequate to stimulate CRC screening adherence. In promoting CRC screening, participants prefer physicians to provide health education. Future interventions will require in-depth understanding of patients’ beliefs, risk perception, and affective responses.

## Author Contribution Statement

Mei Wai Chan, Kooi Yau Chean, Siti Fatimah Kader Maideen and Fei Ping Kow contributed to the design, conduct of the study, analysis of the results and to the writing of the manuscript.

## Funding Statement

This study was funded by RCSI&UCD Malaysia Campus, Penang, Malaysia.

## Ethical consideration

The study was approved by the Medical Research Ethics Committee, Malaysia (NMRR-18-3118-44467) on November 14, 2018. 

## Conflicts of interest

The authors declare no conflict of interest.
